# Study on biomarkers associated with epigenetic factors in endometriosis combining transcriptome with experimental validation

**DOI:** 10.7717/peerj.20703

**Published:** 2026-02-03

**Authors:** Juan Du, Zili Lv, Xia Zheng, Jinpeng Wang, Hua Lu

**Affiliations:** 1 School of Medicine and Life Sciences, Chengdu University of Traditional Chinese Medicine, Chengdu, Sichuan, China; 2 Chengdu University of Traditional Chinese Medicine, Chengdu, Sichuan, China

**Keywords:** Endometriosis, Epigenetic factor, Immune infiltration, Machine learning, Transcriptome

## Abstract

**Background:**

Endometriosis (EM) is a disease related to reproductive dysfunction. The mechanism of epigenetic factors (EF) in EM still needs to be studied. Emerging evidence suggests that EF plays a role in the development of EM. However, the specific molecular pathways through which they exert their effects remain incompletely understood, necessitating further in-depth research. This study aimed to explore the mechanisms underlying EF in EM.

**Methods:**

In the study, the differentially expressed genes (DEGs) between EM and control were obtained by analyzing transcriptome data from public databases. Candidate genes were obtained by taking the intersection of DEGs and EF-related genes (EF-RGs), which were further screened using machine learning algorithms, receiver operating characteristic analysis, and expression levels in the EM and control samples to obtain biomarkers. The potential mechanisms of biomarkers in EF were further analyzed by constructing a nomogram model, gene set enrichment analysis (GSEA), immune infiltration analysis, expression profiling in tissues and cells, molecular regulatory networks, and drug prediction. The expression of these biomarkers was validated using in vitro experiments.

**Results:**

Histone deacetylase 9 (*HDAC9*), YY1-associated factor 2 (*YAF2*), and cell division cycle 6 (*CDC6*) were identified as EF-associated biomarkers in EM. These biomarkers had excellent diagnostic ability for EM. *HDAC9*, *CDC6*, and *YAF2* were respectively significantly enriched in focal adhesion and oxidative phosphorylation pathways. Four types of differentially distributed immune cells were identified between EM and control samples using immune infiltration analysis. The expression of these biomarkers in different tissues varied with age and menstrual cycle. The expression levels of biomarkers were higher in endothelial cells. Ten miRNAs and 24 lncRNAs that targeted these biomarkers were screened, and there were 12 transcription factors (TFs) in which all the biomarkers acted together. All biomarkers worked together for drugs, including bisphenol A, benzo(a)pyrene, and cisplatin. The results of *in vitro* experiments were consistent with those of the bioinformatics analysis.

**Conclusion:**

This study identified three biomarkers (*HDAC9*, *CDC6*, and *YAF2*) and the potential therapeutic drugs for EM. These results provide new insights into the mechanisms underlying EM development.

## Introduction

Endometriosis (EM) is a common benign gynecological condition, primarily distinguished by chronic pain and infertility (Wang, Ji & Sun, 2024). It is characterized by the presence of tissue resembling the endometrium outside the uterine cavity, affecting an estimated 10% of women of reproductive age worldwide (Saunders & Horne, 2021). EM is a common yet frequently overlooked gynecological disorder, contributing to substantial morbidity from adolescence to menopause due to delayed diagnosis and inadequate clinical awareness (Saunders, Whitaker & Horne, 2024). The diagnostic approach typically includes a comprehensive history, pelvic examination, transvaginal ultrasound, magnetic resonance imaging, and laparoscopy (Yoldemir, 2023). However, negative results from these assessments cannot definitively rule out the condition, and delayed treatment can severely impact both quality of life and disease progression (Crump, Suker & White, 2024). Due to the need for surgical confirmation to achieve a definitive diagnosis, EM is often associated with considerable diagnostic delays, impeding timely treatment. Timely and precise clinical evaluation is vital for early detection, facilitating prompt, personalized multidisciplinary interventions and improving therapeutic outcomes (Taylor, Kotlyar & Flores, 2021). Thus, there is a critical need for reliable biomarkers with sufficient sensitivity and specificity to improve the diagnostic process for EM.

Epigenetics, referring to the diverse mechanisms that govern gene regulation and chromatin dynamics, is crucial both for normal development and for the onset and progression of various diseases (Viré & Mead, 2023). In recent years, research on epigenetic factors (EF) has made remarkable progress in elucidating disease mechanisms and driving advancements in precision medicine. Epigenetic regulation influences gene expression through mechanisms such as DNA methylation, histone modifications, and non-coding RNAs without altering the underlying DNA sequence, positioning it as a crucial link between genetic factors, environmental influences, and disease pathogenesis (Farsetti, Illi & Gaetano, 2023). EF refer to molecules that orchestrate epigenetic modifications by modulating DNA and histone marks, facilitating their addition, removal, or recognition, while also governing chromatin remodeling (Marakulina et al., 2023). Epigenetics plays a significant role in the initiation and progression of gynecological cancers. Recent studies have demonstrated that alterations in DNA methylation landscapes, aberrant patterns of specific histone modifications, such as H3K27me3, and dysregulated microRNA expression play essential roles in the pathogenesis of EM (Wang et al., 2022; Adamczyk et al., 2025; Raja et al., 2021). For instance, Chen et al. (2020) reported that the expression of estrogen and progesterone receptors in EM is modulated through epigenetic mechanisms. Nonetheless, most existing investigations focus on isolated epigenetic pathways or individual candidate genes, while comprehensive screening and rigorous validation of key EM-associated epigenetic regulators, and their potential utility as diagnostic biomarkers remain insufficiently explored.

In this study, we integrated transcriptomic datasets with curated resources on epigenetic regulators to systematically uncover epigenetic factor–associated biomarkers relevant to EM. A combination of machine-learning frameworks and experimental validation was employed to refine this set of candidates. Their diagnostic performance was then characterized, followed by an in-depth investigation of their putative molecular roles, their relationships with immune cell infiltration, and their potential connections to therapeutic compounds. The findings presented here not only broaden the current understanding of the epigenetic landscape of endometriosis but also highlight promising noninvasive diagnostic indicators and therapeutic targets. Together, these advances help steer the field toward more precise and individualized approaches to the management of endometriosis.

## Materials & Methods

### Data collection

EM-related transcriptome data (GSE11691 and GSE25628) were downloaded from a database. GSE11691 (GPL96) was used as the training set, which included nine eutopic endometrial tissue (EM) samples and nine *in situ* endometrial tissue (control) samples. GSE25628 (GPL571) was used as the validation set, which included seven eutopic endometrial tissue (EM) samples and nine *in situ* endometrial tissue (control) samples. The 720 EF related genes (EF-RGs) were downloaded from the EpiFactors database (Cheng, Mitra & Coller, 2023) (Table S1).

### Identification of differentially expression genes (DEGs)

The DEGs between EM and control samples in GSE11691 were identified *via* “limma” package (v 3.58.1) (Ritchie et al., 2015). The screening criteria was *P* < 0.05 and —log_2_Fold Change (FC)— > 0.5. The result were shown as volcano plots and heat plots. According to log_2_FC value, the volcano plot displayed DEGs *via* “ggplot2” package (v 3.4.1) with the top 10 up/down-regulated genes labeled, the heat plot displayed the top 10 up/down-regulated genes between EM and control *via* “ComplexHeatmap” package (v 2.20.0) (Gustavsson et al., 2022; Gu, Eils & Schlesner, 2016).

### Identification and function of candidate genes

The candidate genes were obtained by intersection of DEGs and EF-RGs *via* “Venndiagram” package (v 0.1.9) (Gao, Yu & Cai, 2021). Gene Ontology (GO) and Kyoto Encyclopedia of Genes and Genomes (KEGG) were employed to analyze the pathways and biological functions involved in candidate genes *via* “clusterProfiler” package (v 4.7.1.3) (Wu et al., 2021). The screening criteria were *P* < 0.05. GO analysis included biological processes (BP), cellular components (CC), and molecular function (MF). The first three functions with the largest number of enriched genes in each part were shown. The TOP 10 pathways of the KEGG analysis results were displayed according to the *P*-values sorted from smallest to largest. A protein–protein interaction (PPI) network for candidate genes was constructed using the STRING database to analyze the interactions among their encoded proteins. A confidence threshold of 0.4 was set, and isolated nodes were removed before network construction. Subsequently, the remaining genes were imported into the CytoHubba plugin, and the top 20 genes were selected based on the DMNC algorithm for subsequent analysis.

### Identification of biomarkers

The support vector machine recursive feature elimination (SVM-RFE) algorithm, receiver operating characteristic (ROC) analysis, and gene expression level verification were used for further gene screening. Boruta is a feature importance evaluation method based on random forests. It conducts multiple iterative comparisons by constructing shadow features, enabling a comprehensive assessment of each gene’s contribution to classification and avoiding the omission of potentially important features. In GSE11691, the top 20 candidate genes were put into Boruta algorithm using “Boruta” package (v 8.0.0) (*P* = 0.001, maxRuns = 50) (Zhou, Xin & Li, 2023). SVM-RFE shows stable performance in high-dimensional small-sample data. It can effectively handle nonlinear relationships in gene expression data, and gradually optimize feature subsets by recursively eliminating redundant features, thus exhibiting good interpretability and high classification accuracy. The top 20 genes were again put into the SVM-RFE algorithm using “e1071” package (v 1.7.14) (*k* = 5, halve above = 100) (Yang et al., 2022). The genes obtained by the 2 algorithms were intersected to obtain intersection genes *via* “VennDiagram” package (v 0.1.9). These two algorithms complement each other from different perspectives—model accuracy and feature importance—thereby enhancing the reliability of the screening results. ROC analysis was employed to explore the ability of the intersecting genes to distinguish between EM and control samples. In all samples of GSE11691 and GSE25628, the ROC analysis was performed *via* “pROC” package (v 1.18.5) (Robin et al., 2011). The Area Under Curve (AUC) value > 0.8 indicated the gene had a good ability to distinguish the EM and control samples. Genes that passed the ROC analysis were key genes. Verification of gene expression levels was performed to explore the expression levels of key genes in EM and control samples. In GSE11691 and GSE25628, the difference in expression levels between EM and control samples was determined using the Wilcoxon test (*P* < 0.05). Genes with significant differences between EM and control samples and consistent expression trends in both datasets were used as biomarkers.

### Construction of nomogram

A nomogram was employed to explore the diagnostic ability of EM biomarkers. In all samples of GSE11691, the nomogram based on biomarkers was constructed *via* “rms” package (v 6.5.0) (Xu et al., 2023). In the nomogram, biomarkers were pointed separately, each biomarker corresponded to a point, and the points of each biomarker were added together to correspond to the total points. The higher the total number of points, the higher the risk of EM. Calibration curve *via* “calibrate” package (v 1.7.7) (Sanz et al., 2017) and ROC curve *via* “pROC” package (v 1.18.5) was employed to evaluate the accuracy of the nomogram. Calibration curve slope closed to 1 and AUC > 0.7 indicated the nomogram was accurate.

### Gene set enrichment analysis (GSEA)

GSEA was used to analyze the biological functions of the biomarkers. The reference set was “c2.kegg.v7.4.symbols” in the Molecular Signatures Database (MSigDB). In all samples of GSE11691, the Spearman correlation analysis between each biomarker and all the remaining genes was performed *via* “psych” package (v 2.4.3) (Robles-Jimenez et al., 2021). After the correlation coefficients were ranked from greatest to smallest, GSEA was performed *via* “clusterProfiler” package (v 4.7.1.3) (—NES— > 1, FDR < 0.25, *P* < 0.05), and the first 5 results were presented.

### Immune infiltration analysis

Immune infiltration analysis was performed to explore immune cell infiltration in EM. In all samples of GSE11691, the infiltration abundance of 22 immune cells between EM and control samples was performed using the CIBERSORT algorithm (v 0.1.0) and displayed through “ggplot2” package (v 3.4.1), and samples with *P* > 0.05 were excluded (Jiang et al., 2022; Chen et al., 2018). The cell types that were expressed in more than half of the samples were employed to produce the immune cells with significant expression difference between EM and control samples *via* Wilcoxon test (*P* < 0.05), and the result was displayed *via* “ggplot2” package (v 3.4.1). Spearman correlations analysis was employed to explore correlation between differential immune cells or between biomarkers and differential immune cells *via* “psych” package (v 2.4.3) (—cor— > 0.3, *P* < 0.05).

### Expression of biomarkers in tissue and cells

Turku database was established by the University of Turku, and its data are all derived from EM-related samples and matched control samples, which avoids potential biases that may be caused by non-disease-specific datasets. This specialization makes it the optimal tool for verifying the expression patterns of biomarkers in EM-associated tissues. To analyze the expression of biomarkers in different endometrial tissues, different menstrual cycles and ages of EM patients and healthy samples, the clinical differences in each biomarker were determined using the Turku database. As a comprehensive human proteomics resource, the Human Protein Atlas (HPA) provides crucial protein-level validation, which can complement the limitations of the Turku database in transcriptome-level research. To validate the protein expression of biomarkers, determine their spatial localization in target tissues, and lay a foundation for subsequent functional studies and clinical translation, the HPA database was used to measure the expression of biomarkers in different EM cell types.

### Construction of molecular regulatory network and drug prediction

A molecular regulatory network was employed to explore the regulatory relationships between the biomarkers and upstream regulatory molecules.The DIANA-microT, miRanda, TargetScan, PicTar, and microCosm databases were used to predict the miRNAs. The intersection miRNAs from five databases were used for subsequent analysis *via* “Venndiagram” package (v 0.1.9). The StarBase database was used to predict the lncRNAs (clipExpNum > 10). The mRNA-miRNA-lncRNA regulatory relationships were analyzed using a Sankey diagram. The Cistrome database was used to predict TFs. The TF mRNA regulatory network was visualized using Cytoscape (v 3.9.1) (Shannon et al., 2003).

To identify potential therapeutic drugs for EM, a comparative toxicogenomics database (CTD) was used to predict drugs based on biomarkers. Drugs with more than one interaction with the biomarker were used for subsequent network construction and the biomarker-drug network was displayed using Cytoscape (3.9.1).

### Real-time quantitative polymerase chain reaction (RT-qPCR) validation of animal models

A rat model of EM was established using an autologous endometrial transplantation method in specific pathogen-free (SPF) female Sprague-Dawley (SD) rats weighing 190–210 g (Jones, 1984). SD rats were obtained from Chengdu Dashuo Laboratory Animal Co., Ltd. (Production Certificate No. 51203500035896; License No. SCXK (Chuan) 2020-030). Quality control was performed by the supplier. Animals were maintained under SPF conditions with controlled temperature (22 ± 2^∘^C), humidity (55% ± 10%), and a 12 h light/dark cycle. Standard chow and water were provided ad libitum. Environmental enrichment (*e.g.*, nesting materials, exploration objects) was regularly supplied. Randomization was used in the experiment to avoid allocation bias by randomly assigning animals to different experimental (*n* = 5) and control (*n* = 5) groups.

Rats were anesthetized with an intraperitoneal injection of 7% chloral hydrate at a dose of 300mg/kg. Under sterile conditions, the endometrial tissue was excised and trimmed into five mm × five mm fragments. Endometrial fragments were grafted bilaterally *via* musculofascial interface tunnels. Parallel subcutaneous tunnels were created between the abdominal musculature and superficial fascia at the incision flanks, with grafts positioned endometrium-facing the muscle layer. Layered closure was performed using non-absorbable 5-0 nylon sutures. To facilitate lesion development, diethylstilbestrol (0.02 mg/kg) was administered *via* oral gavage for three consecutive days post-transplantation. Lesion progression was monitored every three days by abdominal palpation to assess changes in size and texture. Four weeks after surgery, rats were anesthetized *via* intraperitoneal injection of 7% chloral hydrate (300 mg/kg), followed by laparotomy to assess ectopic lesions, confirming successful model establishment defined by lesion volume enlargement to ≥8 mm^3^, the presence of translucent cystic structures ≥2 mm in diameter containing fluid accumulation, and the formation of a fibrous capsule with visible neovascularization (Fig. S1). Ectopic lesions meeting these criteria were excised under anesthesia induced by intraperitoneal injection of 7% chloral hydrate (300mg/kg) for histopathological confirmation of endometrial origin and subsequent analysis. Meanwhile, normal endometrial tissue from control rats (*n* = 5), anesthetized similarly and subjected to sham surgery (abdominal wall incision only without transplantation), was collected for comparison.

At the end of the experiments, animals were euthanized humanely by an overdose of intraperitoneal chloral hydrate (7%, 500 mg/kg), followed by cervical dislocation to ensure death, in accordance with institutional ethical guidelines.

To verify the accuracy of the bioinformatics analysis results, the difference in biomarkers (*HDAC9, CDC6, and YAF2*) expression between the EM (*n* = 5) and control (*n* = 5) groups was verified by RT-qPCR. This study was approved by the Ethics Committee of the Chengdu University of Traditional Chinese Medicine (2024083). Total RNA was extracted using TRIzol reagent (Ambion, Austin, TX, USA). RNA concentrations were determined using NanoPhotometer N50. Next, mRNA was reverse transcribed into cDNA using the SweScript First Strand cDNA synthesis kit test kit (Servicebio, Wuhan, China). During above process, the reaction conditions (2ug total RNA, 1ul SweScript RT I Enzyme Mix) were as follows: 25 °C for 5 min, 50 °C for 15 min, 85 °C for 5 s, and finally at 4 °C. Finally, RT-qPCR was performed by CFX Connect Real-Time Quantitative Fluorescence PCR Instrument (Bio-Rad, Hercules, CA, USA) and during this process, the requirements for primers could be found in Table S2. The reaction system consists of: three ul of cDNA, five ul of 2x Universal Blue SYBR Green qPCR Master Mix, 1 ul of Forward primer (10 µM), and 1 ul of Reverse primer (10 µM). The reaction temperatures were as follows: pre-denaturation at 95 °C for 1 min, denaturation at 95 °C for 20 s, annealing at 55 °C for 20 s, and extension at 72 °C for 30 s. The expression levels of biomarkers in the EM and control samples were calculated by 2^−ΔΔCt^. *GAPDH* was selected as the internal reference for normalization because of its stable expression, high amplification efficiency, and extensive literature support, enabling experimental standardization and reliable result comparison. *GAPDH* was used as an internal reference to normalize results. The results were calculated using GraphPad Prism version 5.

### Statistical analysis

Bioinformatics analyses were performed using the R programming language (v 4.3.1; R Core Team, 2023). The Wilcoxon test was used to compare differences between the two groups. Statistical significance was set at *P* < 0.05. Differences in expression between EM and control samples were measured using a *t*-test in the RT-qPCR experiment (*P* < 0.05).

**Figure 1:**
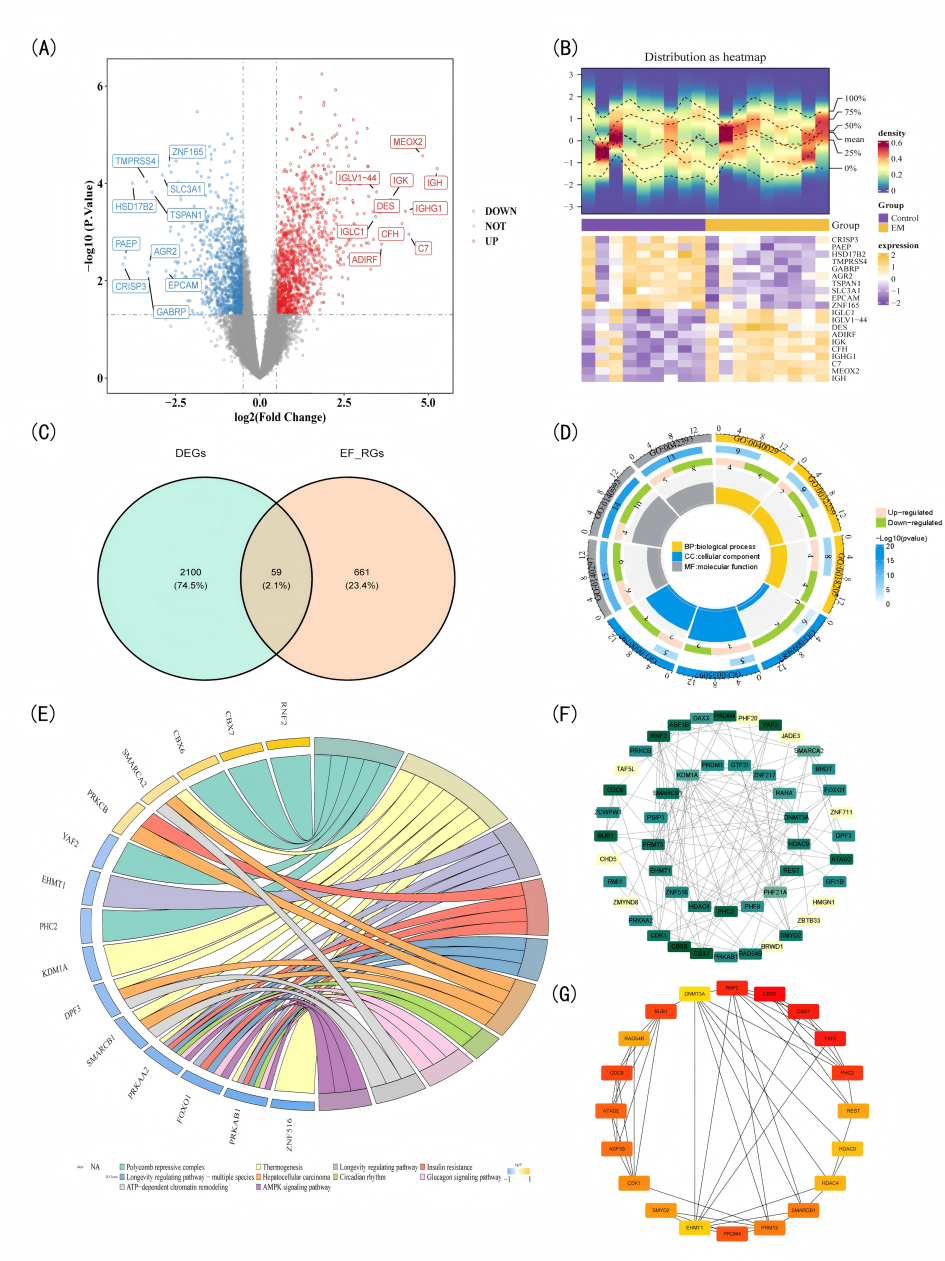
Acquisition of candidate genes and functional enrichment analysis. (A) Volcano plot of differentially expressed genes (DEGs): red dots denote upregulated genes, blue dots indicate downregulated genes; top 10 most significantly altered genes in each direction are labeled. (B) Heatmap of DEGs: the upper panel displays sample expression distribution (blue to red indicates increasing sample abundance), while the lower panel represents gene expression levels (yellow for high expression, purple for low expression). (C) Venn diagram of candidate genes. (D) GO enrichment analysis of candidate genes: outer ring (GO term IDs), middle ring (color intensity denotes significance; bar length and numbers indicate enriched gene counts), inner ring (upregulated in pink, downregulated in green), and central tiles (size reflects RichFactor). (E) KEGG co-enrichment analysis of candidate genes: outer ring (annotated genes, blue for downregulated, yellow for upregulated); top 10 enriched pathways are displayed below. (F) Protein–protein interaction (PPI) network: node color intensity and size reflect potential functional significance, with stronger interactions indicated by darker hues and larger circles. (G) Top 18 genes identified by DMNC algorithm: darker label colors indicate higher gene scores.

## Results

### DEGs and functions and pathways of candidate gene

In the transcriptome dataset GSE11691, compared to the control tissue samples (endometrial), there were 2,159 DEGs in EM tissue samples (endometrial), including 1,133 up-regulated genes and 1,026 down-regulated genes (Table S3). All DEGs were sorted according to the absolute value of log_2_FC, and the top 10 up—regulated genes and the top 10 down—regulated genes with the largest absolute values were selected for drawing a volcano plot (Fig. 1A) and a heat map (Fig. 1B) to show the most significantly DEGs between the EM group and the control group. The aforementioned differences were all based on the detection and analysis of mRNA expression levels. A total of 59 candidate genes were screened by intersecting the 2,159 DEGs with 720 EF-RGs from the EpiFactors database (Fig. 1C). GO analysis of 59 candidate genes enriched 206 functions, including 122 BP, 32 CC, and 52 MF (Table S4). The functions with the highest number of enriched genes included epigenetic regulation of gene expression, methylation, peptidyl-lysine modification, heterochromatin, histone-modifying activity, and histone binding (Fig. 1D). KEGG analysis of 59 candidate genes enriched 130 pathways (Table S5), including thermogenesis, insulin resistance, circadian rhythm, and the AMPK signaling pathway (Fig. 1E). To investigate protein–protein interactions among 59 candidate genes, a PPI network was constructed using the STRING database. After removing 10 isolated nodes, the network comprised 49 proteins encoded by interacting genes. From this network, the top 20 genes were selected *via* the DMNC algorithm for subsequent analysis (Figs. 1F–1G).

### Identification of biomarkers

**Figure 2:**
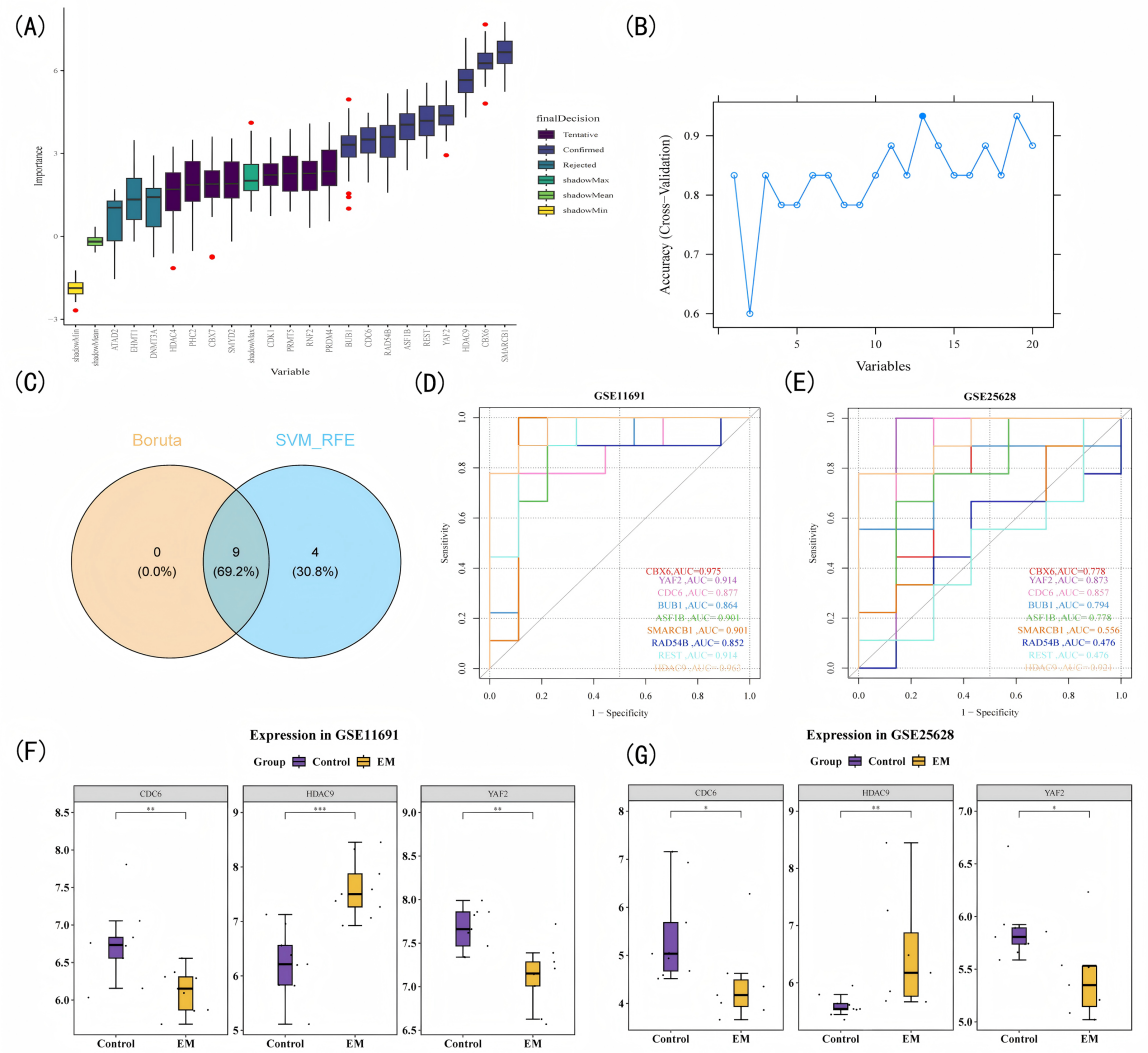
Machine learning-driven biomarker discovery. (A) Boruta feature selection analysis: predictor genes (*x*-axis) *versus* random forest importance scores (*y*-axis). (B) SVM-RFE analysis: feature gene count (*x*-axis) *versus* model prediction accuracy (*y*-axis). (C) Venn diagram illustrating the intersection of feature genes identified by Boruta and SVM-RFE analyses. (D–E) ROC analysis of key genes: false positive rate (1-Specificity) (*x*-axis) *versus* sensitivity (*y*-axis). (F) Expression profiles of pivotal genes in the training cohort. (G) Validation cohort expression profiles of signature genes. *P < 0.05, **P < 0.01, ***P < 0.001.

Based on the top 20 candidate genes, nine genes were identified using the Boruta algorithm and 13 genes were identified using the SVM-RFE algorithm (Figs. 2A–2B). Boruta is a feature importance method based on random forests that comprehensively evaluates the contribution of genes to classification. Finally, nine overlapping genes (*HDAC9*, *YAF2*, *CDC6*, *CBX6*, *BUB1*, *ASF1B*, *SMARCB1*, *RAD54B*, *REST*) were determined by intersecting the genes obtained from the Boruta and SVM-RFE algorithms (Fig. 2C). ROC analysis was performed to evaluate the diagnostic potential of each individual gene in distinguishing EM samples from control samples. The results demonstrated that *HDAC9*, *YAF2*, and *CDC6* each exhibited excellent discriminatory power on their own, with the AUC values for all three genes exceeding 0.8 in both the GSE11691 (*HDAC9*: 0.963, *YAF2*: 0.941, *CDC6*: 0.877) and GSE25628 (*HDAC9*: 0.921, *YAF2*: 0.873, *CDC6*: 0.857) datasets (Figs. 2D–2E). This indicated that each of these genes possessed a good individual ability to distinguish EM from control tissues samples. In GSE11691, the expression levels of *CDC6* and *YAF2* in EM samples were significantly lower than those in control samples, and the expression levels of *HDAC9* in EM samples were significantly higher than those in control samples (Fig. 2F). The results of GSE25628 were consistent with those of GSE11691; therefore, *HDAC9*, *CDC6*, and *YAF2* were considered biomarkers for subsequent analysis (Fig. 2G). The screening of these genes was also based on their association with EM-related biological processes. For example, *HDAC9* has been demonstrated to promote endothelial-mesenchymal transition and cell invasion in other disease contexts (Xu et al., 2022). As a key regulator of DNA replication, *CDC6* expression may be upregulated by the TGF-β signaling pathway, thereby driving abnormal proliferation of ectopic endometrial cells (Young et al., 2014). Although the role of *YAF2* in EM remains unexplored, it is known to interact with YY1 to regulate transcriptional programs such as apoptosis and fate determination (Basu et al., 2014), processes frequently altered in EM.

### Nomogram model

**Figure 3:**
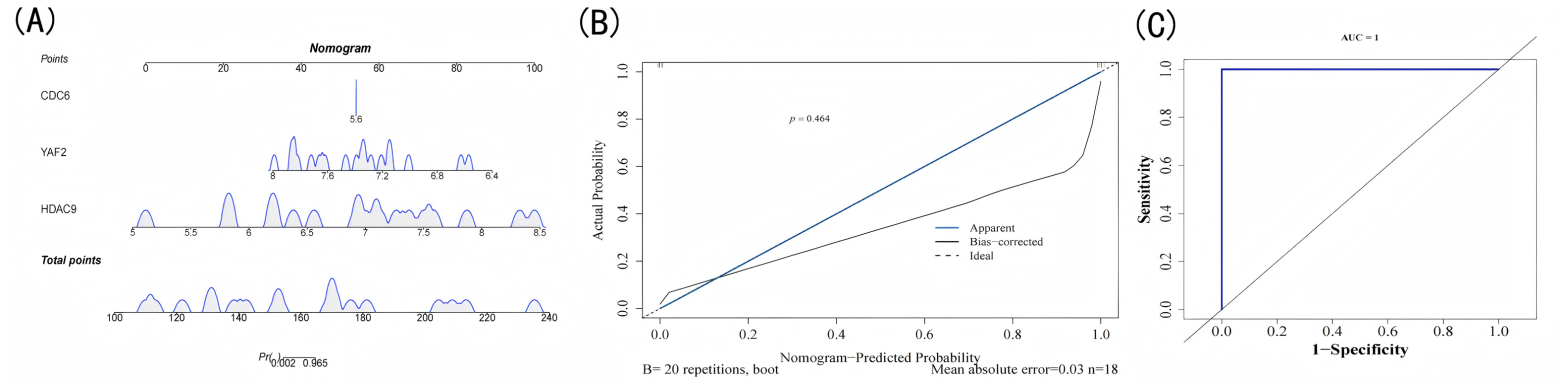
Nomogram construction. (A) Nomogram model: each scale represents a predictor variable, with total points projected onto the outcome probability scale to estimate disease risk. (B) Calibration curve: predicted probabilities (*x*-axis) *versus* observed frequencies (*y*-axis). (C) ROC curve: false positive rate (*x*-axis) *versus* sensitivity (*y*-axis).

A nomogram constructed using *HDAC9*, *CDC6*, and *YAF2* was used to evaluate the diagnostic ability of biomarkers for EM (Fig. 3A). To verify the reliability of the nomogram’s predictive performance, we generated a calibration curve to compare the predicted probability of EM diagnosis derived from the nomogram with the actual clinical diagnosis results of the study cohort. The calibration curve demonstrated an excellent agreement between the nomogram-predicted probability of EM and the actual observed outcome, as the curve closely aligned with the ideal 45-degree reference line (Fig. 3B). The AUC values also verified the accuracy of the nomogram (AUC = 1) (Fig. 3C), which was the maximum value for discriminative ability. It is crucial to note, however, that this result may be attributed to overfitting given the relatively small sample size and should be interpreted with caution. The primary value of this nomogram at the current stage lies in its ability to visually demonstrate the combined diagnostic contribution of the three biomarkers. Its generalizability and clinical utility require further validation in larger, prospective, multi-center cohorts.

### Enrichment pathways of biomarkers

**Figure 4:**
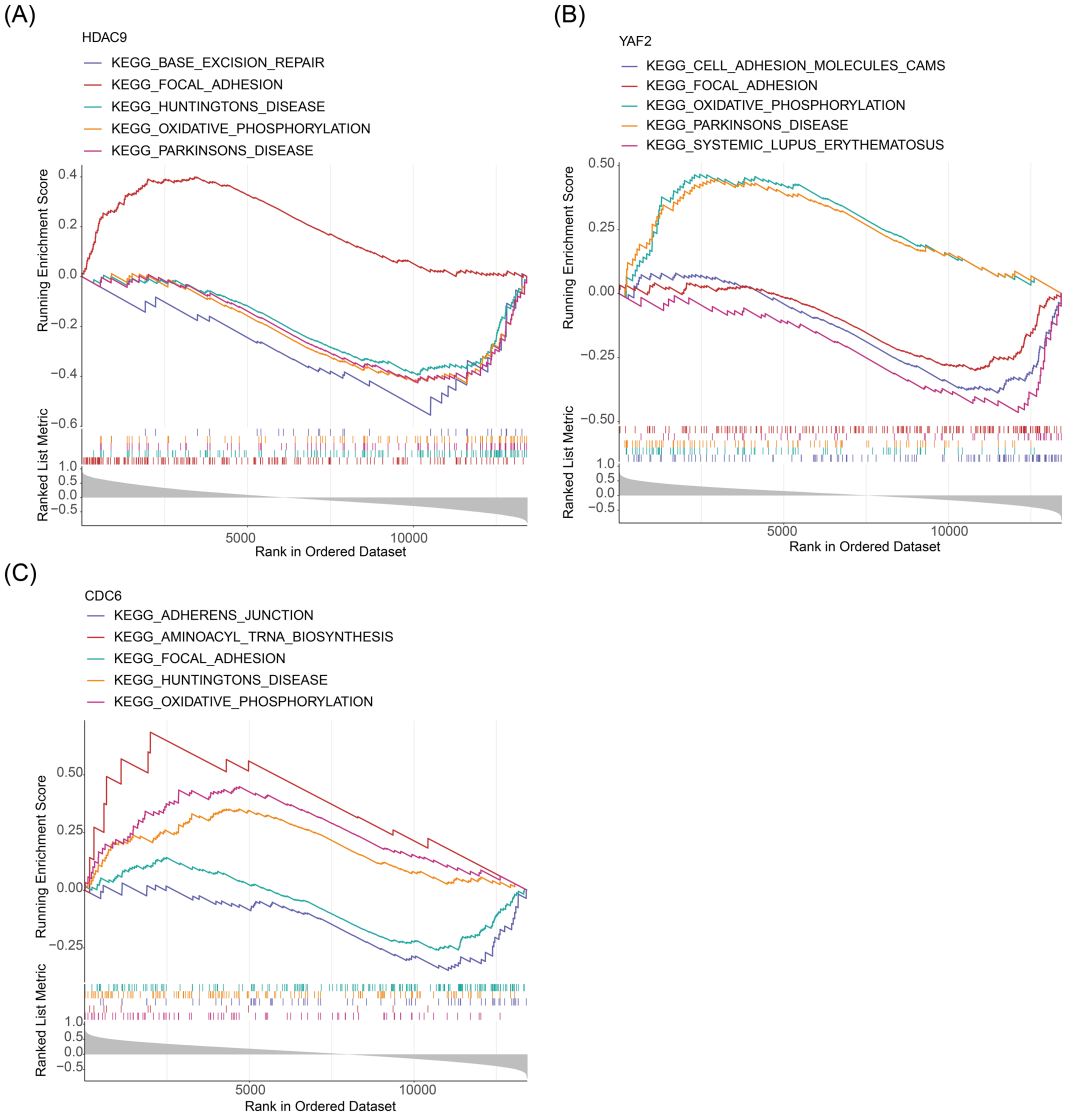
Gene set enrichment (GSEA). (A–C) GSEA of biomarker *HDAC9,YAF2* and *CDC6*.

To elucidate the biological pathways co-regulated with the biomarkers, GSEA was performed based on genome-wide expression correlations. For each biomarker, genes were ranked by their Spearman correlation coefficient with its expression to test for enrichment of predefined KEGG pathways. Using this approach, the expression profile of *HDAC9* was found to be significantly associated with alterations in 27 pathways, including oxidative phosphorylation, focal adhesion, and aminoacyl tRNA biosynthesis (Fig. 4A, Table S6). Similarly, the expression of *YAF2* was significantly enriched in 14 pathways, including oxidative phosphorylation, focal adhesion, and viral myocarditis (Fig. 4B, Table S7). For *CDC6*, its expression pattern was significantly associated with 37 pathways, including focal adhesion, oxidative phosphorylation, glycosphingolipid biosynthesis globo-series, metabolism of xenobiotics by cytochrome P450, and Leishmania infection (Fig. 4C, Table S8). All biomarkers were enriched in the focal adhesion and oxidative phosphorylation pathways.

### Immune cells analysis

**Figure 5:**
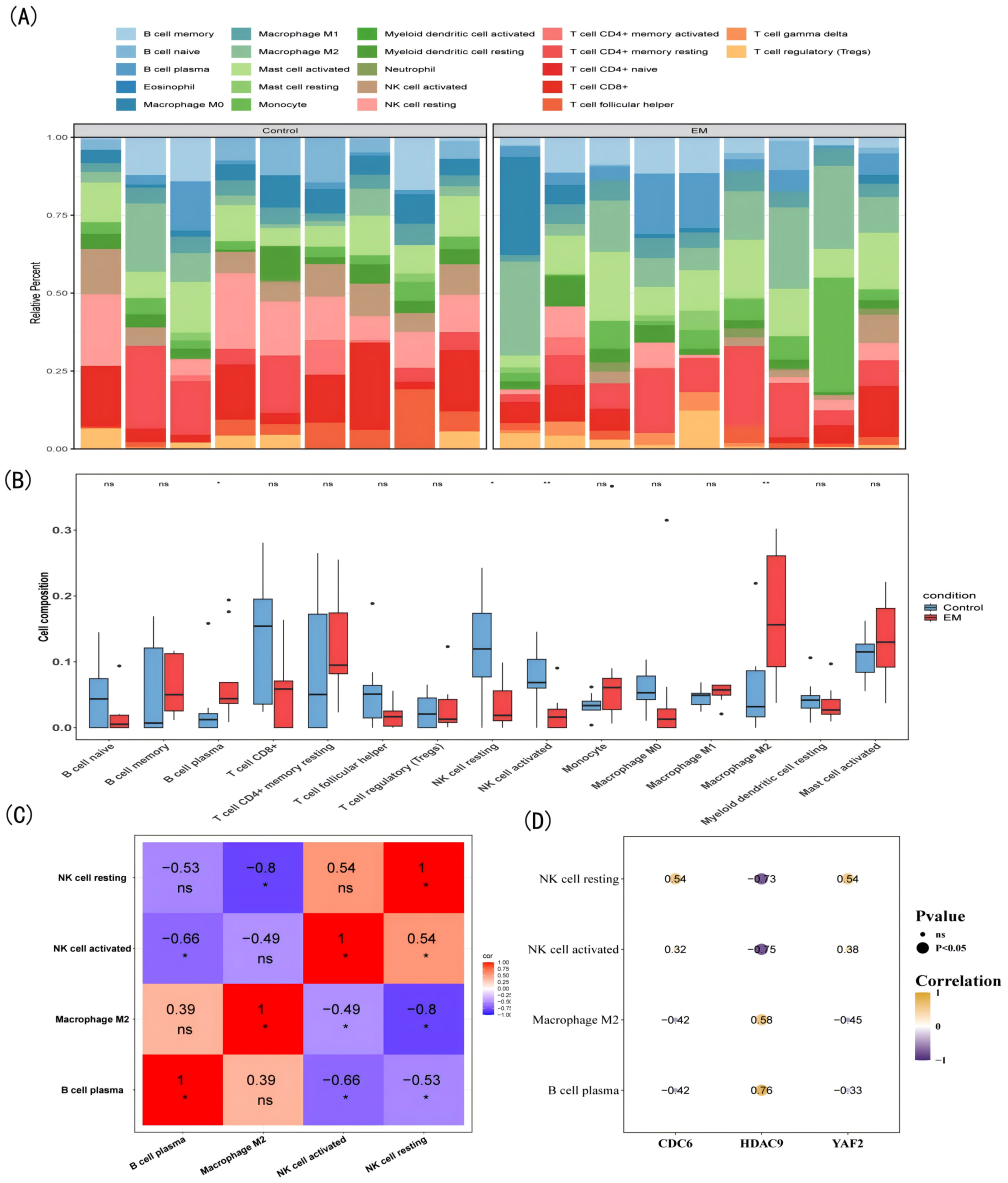
Immune infiltration profiling. (A) Immune cell infiltration profiles: disease *versus* control groups (*x*-axis) *versus* relative abundance (*y*-axis). (B) Infiltration levels of 15 immune cell types (expressed in ≥50% samples) across disease (red) and control (blue) groups. (C) Correlation matrix of differential immune cells: purple to red gradient indicates increasing correlation strength. (D) Correlation between biomarkers and differential immune cells: yellow (positive), purple (negative); circle size denotes significance.

Immune cells played an important role in the occurrence, development and maintenance of EM. To deeply understand the interactions and regulatory mechanisms of immune cells in the immune microenvironment of EM, the abundance of 22 types of immune cells in EM and control samples was determined using the CIBERSORT algorithm (Fig. 5A). The algorithm, based on support vector regression, was applied to deconvolute transcriptomic data using the LM22 signature matrix, quantifying the relative proportions of 22 immune cell types and assessing inter-group differences through 1,000 permutation tests. There were significant differences in the four immune cell types between the EM and control samples, including activated natural killer (NK) cells, M2 macrophages, resting NK cells, and plasma B cells (Fig. 5B). Activated NK cells and resting NK cells had the largest positive correlation (cor = 0.54, *P* < 0.05), whereas M2 macrophages and resting NK cells had the largest negative correlation (cor = −0.80, *P* < 0.05) (Fig. 5C). *HDAC9* had the strongest positive correlation with plasma B cells (cor = 0.76, *P* < 0.05) and the strongest negative correlation with activated NK cells (cor = −0.75, *P* < 0.05) (Fig. 5D). This result provided support for the hypothesis that biomarkers might regulate the progression of EM by modulating immune cell infiltration.

### Analysis of biomarker expression in tissues and cells

Biomarker expression was validated using two complementary databases: the Turku database, which provides endometriosis-specific transcriptomic data to minimize disease-unrelated bias, and the HPA, which offers corroborative protein-level evidence (Fig. S2). Analysis of the Turku database revealed distinct expression patterns of the three biomarkers across tissues, age, and menstrual cycles (Fig. S2A–S2C). In deep infiltrating endometriosis (DIE) lesions, *HDAC9* and *YAF2* were upregulated, while *CDC6* was downregulated, suggesting their combined potential as a diagnostic panel for non-invasive DIE detection (Fig. S2A). This tissue-specific pattern was consistent in eutopic endometrium and peritoneum, where *HDAC9* and *YAF2* were generally elevated, and *CDC6* was reduced in patients compared to controls (Fig. S2A). Given that DIE is associated with severe pain and functional impairment, and its non-invasive diagnosis remains challenging (Zamurovic et al., 2023), this specific signature presented significant clinical value. Our study suggested that the combined detection of these three genes could form a high-accuracy, non-invasive diagnostic model, thereby offering a new strategy to address this pressing unmet clinical need. Expression varied with age and menstrual phase: *HDAC9* was lowest in the 30–39 age group but overall higher in EM tissues; *CDC6* fluctuated with age but remained generally low in EM; and *YAF2* increased with age in peritoneal tissues but decreased in ovaries, while being overall high in EM (Fig. S2B). Across the menstrual cycle, *HDAC9* was low in the proliferative phase, *CDC6* was low in the secretory phase, and *YAF2* was high in both phases (Fig. S2C). At the cellular level, *HDAC9* was highly expressed in endothelial, glandular, and luminal cells; *CDC6* in glandular, luminal, and stromal cells; and *YAF2* predominantly in stromal cells (Fig. S2D–S2F).

### Molecular regulation analysis and drug prediction

**Figure 6:**
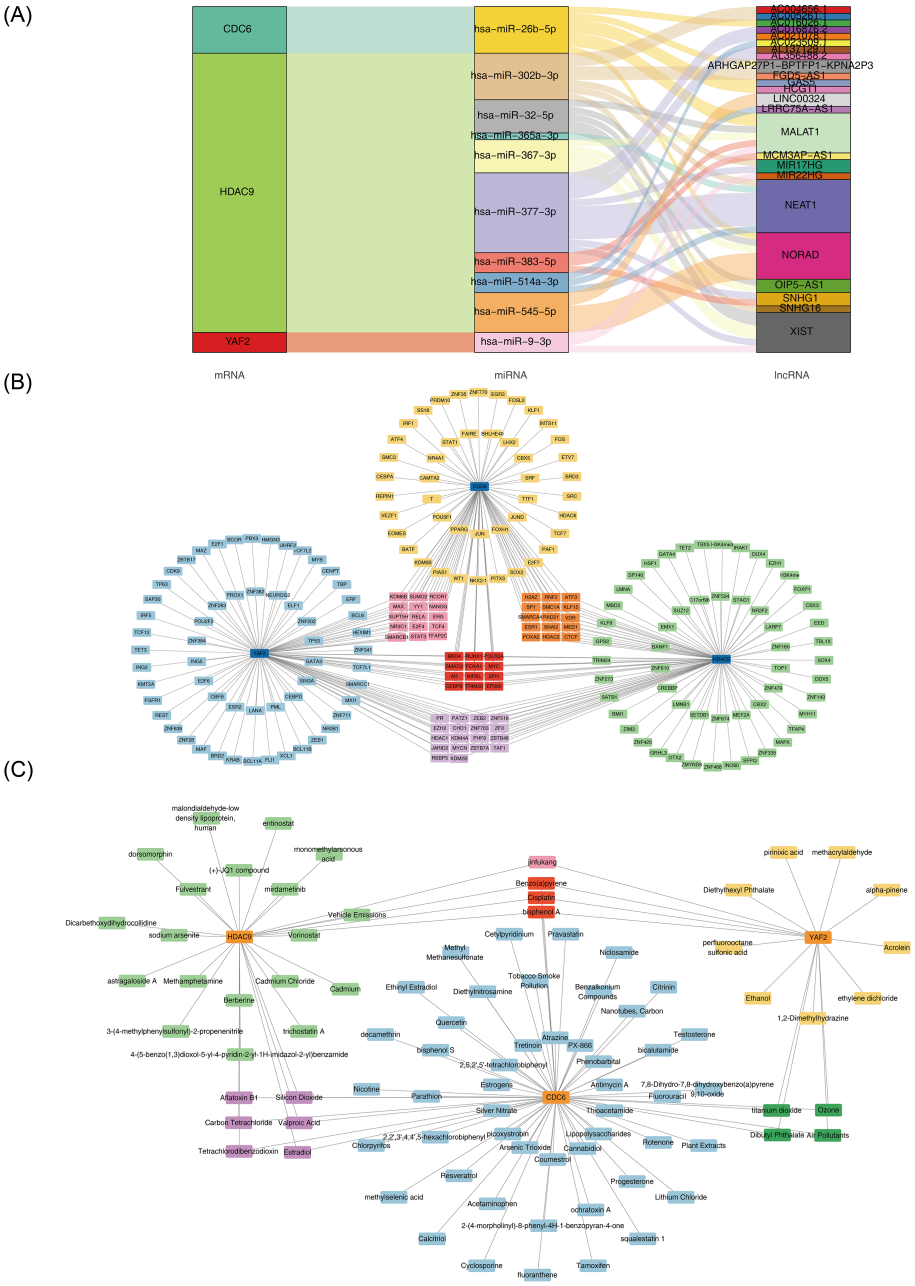
Molecular regulatory network reconstruction. (A) Molecular regulatory network: mRNA (left), miRNA (center), lncRNA (right); connecting lines represent regulatory interactions. (B) TF-biomarker regulatory network: light blue (genes), blue/green/yellow (*YAF2*/*HDAC9*/*CDC6* -targeting TFs), purple/orange/pink (shared TFs). (C) Biomarker-drug interaction network: orange (genes), blue/green/yellow (*CDC6*/*HDAC9*/*YAF2*-targeting drugs), purple/cyan/pink (shared candidate drugs).

The 10 miRNA and 24 lncRNAs were obtained from databases, and the relationships among biomarkers, miRNAs and lncRNAs were displayed through a Sankey diagram, such as *CDC6* targeted hsa-miR-26b-5p, hsa-miR-26b-5p targeted AC016026.1 (Fig. 6A). The TF-biomarker network indicated *CDC6* predicted 87 TFs, *HDAC9* predicted 100 TFs, *YAF2* predicted 103 TFs. There were 12 TFs in which all biomarkers acted together, including BRD4, RUNX1, POLR2A and AR (Fig. 6B). A total of 115 potential drugs for *HDAC9*, 205 potential drugs for *CDC6*, and 66 potential drugs for *YAF2* were predicted using the CTD database. The biomarker-drug networks identified 29, 62, and 17 potential drugs targeting *HDAC9*, *CDC6*, and *YAF2*, respectively. Notably, all three biomarkers were predicted to potentially interact with bisphenol A, benzo[a]pyrene, and cisplatin (Fig. 6C, Table S9). However, the clinical application of these drugs in EM remains to be experimentally verified.

### RT-qPCR analysis

**Figure 7:**
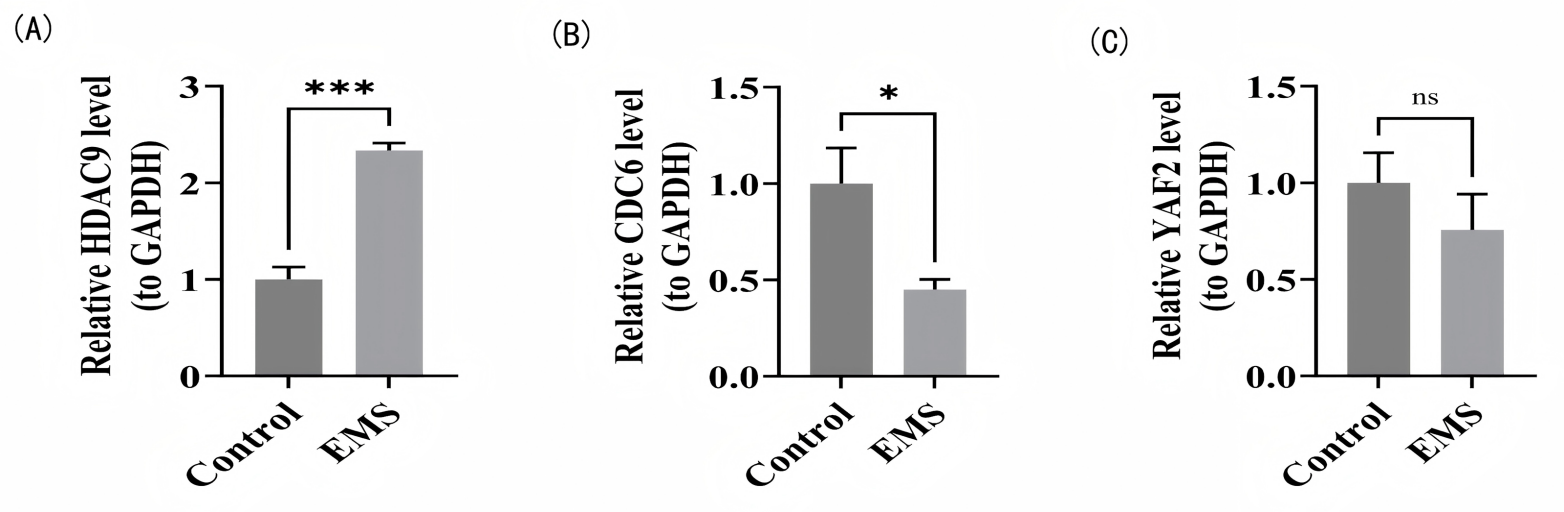
RT-qPCR validation of biomarker expression. (A) *HDAC9* mRNA in EM lesions *vs.* controls. (B) *CDC6* mRNA in EM lesions *vs.* controls. (C) *YAF2* mRNA in EM lesions *vs.* controls. *P < 0.05, ***P < 0.001.

The relative mRNA expression levels of EM and the control were determined by RT-qPCR. The results showed the *HDAC9* expression levels were significantly higher in the EM group than in the control group (*P* < 0.001), whereas *CDC6* expression levels were significantly lower in the EM group than in the control group (*P* < 0.05) (Figs. 7A–7B). The *YAF2* expression levels were lower than those in the control group, but there were no significantly differences between the EM and control groups (Fig. 7C). This discrepancy between the bioinformatic prediction and experimental validation for *YAF2* could be attributed to several factors, including but not limited to: (1) potential post-transcriptional or post-translational regulation that masks mRNA-level changes; (2) the limited sample size in our animal model, which might have reduced the statistical power to detect a subtle but real difference; or (3) species-specific differences between the human transcriptome data and the rat model. Nonetheless, the expression trends of all three biomarkers were consistent with the bioinformatics analysis.

## Discussion

EM is a common gynecological disorder characterized by symptoms such as dysmenorrhea, infertility, painful bowel movements, and pelvic pain, which significantly impact patients’ quality of life and pose a severe threat to women’s overall health (Saunders & Horne, 2021). A research has highlighted a relationship between EF and EM (Throwba et al., 2022). However, the precise mechanisms by which EF contribute to EM remain unclear. Therefore, identifying potential biomarkers and elucidating the molecular mechanisms involved in EM are essential for improving the diagnostic accuracy and developing effective therapeutic strategies (Jiang et al., 2022).

In this study, the intersection of DEGs and EF-RGs identified 59 EF-related candidate genes associated with EM. Three biomarkers (*HDAC9*, *CDC6*, and *YAF2*) were identified using machine learning, ROC analysis, and expression validation. GSEA revealed the pathways enriched with these biomarkers. Immune infiltration analysis of the dataset samples identified four EF-related differentially abundant immune cells. Additionally, the expression patterns of the biomarkers across various tissues and cell types were examined. Database predictions identified 10 miRNAs, 24 lncRNAs, 100 TFs targeting *HDAC9*, 87 TFs regulating *CDC6*, 103 TFs modulating *YAF2*, 12 TFs interacting with all biomarkers, and 3 drugs potentially influencing their function.

Histone deacetylase 9 (*HDAC9*), a member of the HDAC family and a histone deacetylase, has been shown to promote endothelial-to-mesenchymal transition upon overexpression, thereby altering gene and protein expression, and potentially influencing cellular behavior and tissue remodeling through epigenetic mechanisms in the pathogenesis of certain diseases (Lecce et al., 2021). Research evidence indicates that other members of the HDAC family, such as *HDAC1*, *HDAC3*, and *HDAC6*, exhibit significant dysregulated expression in EM. For instance, *HDAC3* is downregulated in patients with EM and is closely associated with infertility, as its loss impairs decidualization and leads to implantation failure (Adamczyk, Wender-Ozegowska & Kedzia, 2022). In contrast, *HDAC1* is significantly upregulated in endometriotic lesions and is implicated in disease progression.Similarly, *HDAC6* expression correlates with the severity of endometriotic lesions (Zheng, Liu & Guo, 2023). Current evidence indicates that *HDAC9* exists in two isoforms in endometrial cells: a full-length form and a truncated variant lacking the C-terminal deacetylase domain, the latter of which is highly expressed in breast cancer cells (Gujral, Mahajan & Ponnampalam, 2022). Notably, its elevated expression in breast cancer has been linked to increased tumor invasiveness and enhanced angiogenic potential—biological characteristics that are also observed in EM (Salgado et al., 2018). Epigenetic modifications, including DNA methylation, histone alterations, and microRNA expression, have been implicated in the pathogenesis of endometriosis (Hsiao, Wu & Tsai, 2017). For instance, Lin et al. (2024) demonstrated that m6A RNA methylation and EZH2/H3K27me3-mediated histone methylation can act synergistically to impair decidualization, which may partly explain the infertility observed in patients with endometriosis. However, no existing studies have specifically investigated the role of *HDAC9* in EM. This study provides the first evidence that *HDAC9* plays a role in the epigenetic regulation of EM.

This study also found a decrease in the expression of *CDC6* in EM. Cell division cycle 6 (*CDC6*), a member of the AAA+ ATPase family, serves as a key regulator of DNA replication and cell cycle checkpoints within the pre-replication complex, while also facilitating protein complex dynamics, and is critically involved in cancer development and progression (Li et al., 2025). In EM, the proliferation and invasiveness of ectopic endometrial tissue are prominent pathological characteristics (Takeuchi, Matsuzaki & Harada, 2024). Research indicates that molecules like AC002454.1 and CDK6 act synergistically to promote the migration and invasion of endometrial cells (Liu et al., 2019). A study indicates that CDK6 overexpression may impair *CDC6* function by promoting its degradation or inhibition, potentially through phosphorylation, thereby suppressing DNA replication (Yu et al., 2019). Therefore, we speculate that the down-regulation of CDC6 may indirectly affect DNA replication, thereby promoting the proliferation and invasion of ectopic endometrial tissue. However, this hypothesis awaits subsequent experimental verification.

As an interaction partner of Yin Yang 1 (YY1), Yin Yang 1 Associated Factor 2 (*YAF2*) is implicated in the pathogenesis of EM through its central roles in apoptosis regulation, epigenetic modifications, and YY1-dependent transcriptional control (Li, Ma & Chen, 2016; Basu et al., 2014). This involvement aligns closely with key pathological features of EM, such as aberrant survival of ectopic tissues. *YAF2* may modulate the dysregulated apoptotic processes in EM by stabilizing the pro-apoptotic protein PDCD5, thereby enhancing p53-mediated inhibition of cell proliferation (Geng et al., 2022). Concurrently, *YAF2* exhibits pro-survival activity: in tumor cells, it can exert anti-apoptotic effects *via* FANK1-dependent and phosphorylation-dependent pathways (Zhang et al., 2021), a function that may be conserved in EM and contribute to the evasion of apoptosis by ectopic endometrial cells.

At the epigenetic level, YAF2 participates not only in histone modifications, such as H2AK119 ubiquitination, but also integrates signaling with YY1 to potentiate YY1-mediated transcriptional regulation (Rose et al., 2016). Furthermore, YAF2 can cooperate with other transcription factors or epigenetic regulators to influence cell fate decisions, including stem cell differentiation, highlighting its potential as a therapeutic target in EM (Sawa et al., 2002; Yan et al., 2021). Additionally, YAF2 mediates the interaction between YY1 and sirtuin 6 (SIRT6) to regulate mitochondrial function in senescent cell membranes (Kawamura, Higuchi & Fujiwara, 2021). This mechanism may facilitate the survival of ectopic endometrial cells under the adverse microenvironment of EM, such as hypoxia and nutrient deprivation, thereby indirectly promoting disease progression.

EM is increasingly recognized as an autoimmune-related disorder, and studies have indicated that immune cells such as neutrophils, macrophages, NK cells, and dendritic cells, may play distinct roles in angiogenesis, tissue growth, and the invasion of endometrial-derived cells (Abramiuk et al., 2022; Shin et al., 2023). The immune infiltration analysis in this study revealed significant differences in the immune microenvironment of EM, particularly in activated NK cells, M2 macrophages, resting NK cells, and plasma cells. Notably, plasma cells and resting NK cells exhibited the strongest correlations with the key biomarkers. Plasma cell infiltration in EM, associated with abnormal immune activation and increased B-lymphocyte stimulators, is believed to contribute to the disease’s pathogenesis (Chang & Miao, 2024). Additionally, plasmacytoid dendritic cells (pDCs) in EM patients exhibit increased expression of PD-L1 and PD-L2, highlighting their potential role in immune regulation and tumorigenesis, and plasma cells may influence the immune microenvironment by modulating macrophage function and other immune cell interactions, thereby promoting disease progression (Laganà et al., 2023). Cytokines and chemokines released by plasma cells may contribute to disease progression by influencing epigenetic modifications, such as DNA methylation and histone modifications, within the local microenvironment (Hsiao, Wu & Tsai, 2017).

NK cells are critical immune effectors with cytotoxic capabilities, enabling them to identify and eliminate tumor cells or virus-infected cells (Jiang et al., 2024). However, in patients with EM, NK cell function is markedly impaired, characterized by reduced activity and weakened cytotoxicity. This dysfunction is likely associated with the immunosuppressive microenvironment within endometriotic lesions (Ściezyńska et al., 2019). Resting NK cells, predominantly residing in peripheral blood, the spleen, and bone marrow, remain inactive without cytokine stimulation, such as IL-2 or IL-15, and are characterized by low expression of activation markers like CD69 and CD25, while retaining high surface levels of CD16 (Fc *γ* RIII) (Giansanti et al., 2023). A study has shown that NK cells in EM patients exhibit significantly increased CD94/NKG2A expression and a reduced proportion of CD56dimCD16+ NK cells, indicating impaired resting NK cell function (Yang et al., 2024). Additionally, their cytotoxic activity is diminished, preventing the effective clearance of ectopic endometrial tissue, thereby facilitating its implantation and growth (Li et al., 2023).

By analyzing publicly available datasets, this study uncovered three EF-related biomarkers involved in EM progression that could serve as potential diagnostic markers for distinguishing EM and have clinical relevance. However, because the samples in this study were sourced from tissues affected by EM, the applicability of these biomarkers for early-stage diagnosis may be limited. Future studies should prioritize validating these biomarkers in the blood or other biofluid samples to assess their potential for early detection. This study has several limitations. First, the sample size in the public datasets was relatively small, and in our PCR experiments, the expression of *YAF2* did not show significant differences between samples, which may be attributed to both the limited sample size and considerable inter-sample heterogeneity. Future studies should aim to include larger cohorts and further validate the findings in human clinical samples. In addition, incorporating intermediate experimental models, such as cellular assays, could help bridge the gap between human data and animal studies, thereby enhancing the reliability, generalizability, and translational potential of the conclusions. We will also continue to investigate the mechanisms driving these biomarkers to facilitate more comprehensive and detailed research in the future.

## Conclusions

This study identified three EF-associated biomarkers(*HDAC9*, *CDC6*, and *YAF2*) in EM and nomogram analysis demonstrated their robust diagnostic potential. Subsequent bioinformatic investigations have elucidated the molecular mechanisms underlying the involvement of these biomarkers in EM pathogenesis. Experimental validation using *in vitro* models confirmed the computational predictions and revealed the functional roles of biomarkers in disease progression. The discovery of these molecular signatures and mechanistic insights provides novel perspectives for advancing diagnostic strategies and therapeutic interventions in EM management.

## Supplemental Information

10.7717/peerj.20703/supp-1 Supplemental Information 1 EM rat model establishment (A) Abdominal wall endometriotic lesions of model group rats. (B) Abdominal wall incisions without ectopic lesion of control group rats.

10.7717/peerj.20703/supp-2 Supplemental Information 2 Comparative biomarker distribution among distinct tissue types (A) Expression profiles of biomarkers across endometrial (disease/control), peritoneal (disease/control), and ovarian tissues: tissue status (x-axis) versus expression levels (y-axis, top to bottom: *HDAC9, CDC6, YAF2*). (B) Expression patterns of key genes across age-stratified tissue groups (x-axis: five tissue types) versus expression levels (y-axis, top to bottom: *HDAC9, CDC6, YAF2*). (C) Expression of biomarkers (top to bottom: *HDAC9, CDC6, YAF2*) across cell types, with darker shades indicating higher expression levels. (D–F) Cellular expression profiles of biomarkers *HDAC9, CDC6*, and *YAF2*.

10.7717/peerj.20703/supp-3 Supplemental Information 3 The epigenetic factor-related genes (EF-RGs) from the EpiFactors database

10.7717/peerj.20703/supp-4 Supplemental Information 4 Primer Requirements for RT-qPCR

10.7717/peerj.20703/supp-5 Supplemental Information 5 The differentially expressed genes (DEGs) in EM samples compared with controls

10.7717/peerj.20703/supp-6 Supplemental Information 6 Gene Ontology (GO) enrichment analysis of DEGs

10.7717/peerj.20703/supp-7 Supplemental Information 7 KEGG pathway enrichment analysis results

10.7717/peerj.20703/supp-8 Supplemental Information 8 The significantly enriched KEGG pathways in *HDAC9*

10.7717/peerj.20703/supp-9 Supplemental Information 9 The significantly enriched KEGG pathways in *YAF2*

10.7717/peerj.20703/supp-10 Supplemental Information 10 The significantly enriched KEGG pathways in *CDC6*

10.7717/peerj.20703/supp-11 Supplemental Information 11 The biomarker-specific drug prediction network and shared therapeutic targets

10.7717/peerj.20703/supp-12 Supplemental Information 12 Graphical Abstract Visually illustrates the technical workflow of the present study. Candidate genes were screened by integrating the analysis of GEO dataset (GSE11691) and mining of the EpiFactors database; key genes were then identified following validation via machine learning and the GSE25628 dataset. Subsequent analyses (including nomogram modeling, immune infiltration, and drug prediction) were conducted, with final verification completed using a rat model.

10.7717/peerj.20703/supp-13 Supplemental Information 13 ARRIVE 2.0 Checklist

10.7717/peerj.20703/supp-14 Supplemental Information 14 MIQE checklist

10.7717/peerj.20703/supp-15 Supplemental Information 15 Raw Data of PCR

10.7717/peerj.20703/supp-16 Supplemental Information 16 Code

## Abbreviations

AUC: Area Under Curve
BP: biological processes
CC: cellular components
CDC6: Cell division cycle 6
CTD: comparative toxicogenomics database
DEGs: differentially expressed genes
EF: epigenetic factors
EF-RGs: EF-related genes
EM: endometriosis
ER: estrogen receptor
FC: Fold Change
GO: Gene Ontology
GSEA: gene set enrichment analysis
HDAC9: histone deacetylase 9
HPA: Human Protein Atlas
KEGG: Kyoto Encyclopedia of Genes and Genomes
MF: molecular function
MSigDB: Molecular Signatures Database
NK: natural killer
pDCs: plasmacytoid dendritic cells
PPI: protein–protein interaction
PR: progesterone receptor
ROC: receiver operating characteristic
RT-qPCR: real-time quantitative polymerase chain reaction
SD: Sprague-Dawley
SPF: specific pathogen-free
SVM-RFE: support vector machine recursive feature elimination
TFs: transcription factors
YAF2: YY1 associated factor 2
YY1: YinYang 1

## Additional Information and Declarations

### Microarray Data Deposition

The following information was supplied regarding the deposition of microarray data: The datasets are available at GEO: GSE11691 and GSE25628.

### Data Availability

The following information was supplied regarding data availability: The raw data is available in the Supplemental Files.

## References

[ref-1] Abramiuk M, Grywalska E, Małkowska P, Sierawska O, Hrynkiewicz R, Niedźwiedzka-Rystwej P (2022). The role of the immune system in the development of endometriosis. Cell.

[ref-2] Adamczyk M, Rawłuszko-Wieczorek A, Wirstlein P, Nowacka M, Nowicki M, Jagodziński PP, Wender-Ozegowska E, Kędzia M (2025). H3K27me3-mediated epigenetic regulation of TET1 in the eutopic endometrium of women with endometriosis and infertility. Scientific Reports.

[ref-3] Adamczyk M, Wender-Ozegowska E, Kedzia M (2022). Epigenetic factors in eutopic endometrium in women with endometriosis and infertility. International Journal of Molecular Sciences.

[ref-4] Basu A, Wilkinson FH, Colavita K, Fennelly C, Atchison ML (2014). YY1 DNA binding and interaction with YAF2 is essential for Polycomb recruitment. Nucleic Acids Research.

[ref-5] Chang X, Miao J (2024). Identification of a disulfidptosis-related genes signature for diagnostic and immune infiltration characteristics in endometriosis. Scientific Reports.

[ref-6] Chen B, Khodadoust MS, Liu CL, Newman AM, Alizadeh AA (2018). Profiling tumor infiltrating immune cells with CIBERSORT. Methods in Molecular Biology.

[ref-7] Chen H, Malentacchi F, Fambrini M, Harrath AH, Huang H, Petraglia F (2020). Epigenetics of estrogen and progesterone receptors in endometriosis. Reproductive Sciences.

[ref-8] Cheng MW, Mitra M, Coller HA (2023). Pan-cancer landscape of epigenetic factor expression predicts tumor outcome. Communications Biology.

[ref-9] Crump J, Suker A, White L (2024). Endometriosis: a review of recent evidence and guidelines. The Australian Journal of General Practice.

[ref-10] Farsetti A, Illi B, Gaetano C (2023). How epigenetics impacts on human diseases. European Journal of Internal Medicine.

[ref-11] Gao CH, Yu G, Cai P (2021). ggVennDiagram: an intuitive, easy-to-use, and highly customizable R package to generate venn diagram. Frontiers in Genetics.

[ref-12] Geng Z, Chen H, Zou G, Yuan L, Liu P, Li B, Zhang K, Jing F, Nie X, Liu T, Zhang B (2022). Human amniotic fluid mesenchymal stem cell-derived exosomes inhibit apoptosis in ovarian granulosa cell *via* miR-369-3p/YAF2/PDCD5/p53 pathway. Oxidative Medicine and Cellular Longevity.

[ref-13] Giansanti M, Theinert T, Boeing SK, Haas D, Schlegel PG, Vacca P, Nazio F, Caruana I (2023). Exploiting autophagy balance in T and NK cells as a new strategy to implement adoptive cell therapies. Molecular Cancer.

[ref-14] Gu Z, Eils R, Schlesner M (2016). Complex heatmaps reveal patterns and correlations in multidimensional genomic data. Bioinformatics.

[ref-15] Gujral P, Mahajan V, Ponnampalam A (2022). HDAC class IIa expression and regulation in human endometrial tissues and stromal cells during the menstrual cycle. Research Square.

[ref-16] Gustavsson EK, Zhang D, Reynolds RH, Garcia-Ruiz S, Ryten M (2022). *ggtranscript*: an R package for the visualization and interpretation of transcript isoforms using ggplot2. Bioinformatics.

[ref-17] Hsiao KY, Wu MH, Tsai SJ (2017). Epigenetic regulation of the pathological process in endometriosis. Reproductive Medicine and Biology.

[ref-18] Jiang P, Jing S, Sheng G, Jia F (2024). The basic biology of NK cells and its application in tumor immunotherapy. Frontiers in Immunology.

[ref-19] Jiang H, Zhang X, Wu Y, Zhang B, Wei J, Li J, Huang Y, Chen L, He X (2022). Bioinformatics identification and validation of biomarkers and infiltrating immune cells in endometriosis. Frontiers in Immunology.

[ref-20] Jones RC (1984). The effect of a luteinizing hormone releasing hormone (LRH) agonist (Wy-40, 972), levonorgestrel, danazol and ovariectomy on experimental endometriosis in the rat. Acta Endocrinologica.

[ref-21] Kawamura K, Higuchi T, Fujiwara S (2021). YAF2-mediated YY1-Sirtuin6 interactions responsible for mitochondrial downregulation in aging tunicates. Molecular and Cellular Biology.

[ref-22] Laganà AS, Ferrari F, Mangione D, Fiorino F, Vassiliadis A, Venezia R (2023). Molecular and cellular advances in endometriosis research: paving the way for future directions. International Journal of Molecular Sciences.

[ref-23] Lecce L, Xu Y, V’Gangula B, Chandel N, Pothula V, Caudrillier A, Santini MP, d’Escamard V, Ceholski DK, Gorski PA, Ma L, Koplev S, Bjørklund MM, Björkegren JL, Boehm M, Bentzon JF, Fuster V, Kim HW, Weintraub NL, Baker AH, Bernstein E, Kovacic JC (2021). Histone deacetylase 9 promotes endothelial-mesenchymal transition and an unfavorable atherosclerotic plaque phenotype. Journal of Clinical Investigation.

[ref-24] Li W, Lin A, Qi L, Lv X, Yan S, Xue J, Mu N (2023). Immunotherapy: a promising novel endometriosis therapy. Frontiers in Immunology.

[ref-25] Li G, Ma D, Chen Y (2016). Cellular functions of programmed cell death 5. Biochimica Et Biophysica Acta/General Subjects.

[ref-26] Li C, Zhang ED, Yu R, Yuan B, Yang Y, Zeng Z, Huang H (2025). Comprehensive multi-omics analysis showed that CDC6 is a potential prognostic and immunotherapy biomarker for multiple cancer types including HCC. Translational Oncology.

[ref-27] Lin X, Dai Y, Gu W, Zhang Y, Zhuo F, Zhao F, Jin X, Li C, Huang D, Tong X, Zhang S (2024). The involvement of RNA N6-methyladenosine and histone methylation modification in decidualization and endometriosis-associated infertility. Clinical and Translational Medicine.

[ref-28] Liu J, Wang Y, Chen P, Ma Y, Wang S, Tian Y, Wang A, Wang D (2019). AC002454.1 and CDK6 synergistically promote endometrial cell migration and invasion in endometriosis. Reproduction.

[ref-29] Marakulina D, Vorontsov IE, Kulakovskiy IV, Lennartsson A, Drabløs F, Medvedeva YA (2023). EpiFactors 2022: expansion and enhancement of a curated database of human epigenetic factors and complexes. Nucleic Acids Research.

[ref-30] R Core Team (2023). https://www.r-project.org.

[ref-31] Raja MHR, Farooqui N, Zuberi N, Ashraf M, Azhar A, Baig R, Badar B, Rehman R (2021). Endometriosis, infertility and MicroRNA’s: a review. Journal of Gynecology Obstetrics and Human Reproduction.

[ref-32] Ritchie ME, Phipson B, Wu D, Hu Y, Law CW, Shi W, Smyth GK (2015). *limma* powers differential expression analyses for RNA-sequencing and microarray studies. Nucleic Acids Research.

[ref-33] Robin X, Turck N, Hainard A, Tiberti N, Lisacek F, Sanchez JC, Müller M (2011). pROC: an open-source package for R and S+ to analyze and compare ROC curves. BMC Bioinformatics.

[ref-34] Robles-Jimenez LE, Aranda-Aguirre E, Castelan-Ortega OA, Shettino-Bermudez BS, Ortiz-Salinas R, Miranda M, Li X, Angeles-Hernandez JC, Vargas-Bello-Pérez E, Gonzalez-Ronquillo M (2021). Worldwide traceability of antibiotic residues from livestock in wastewater and soil: a systematic review. Animals.

[ref-35] Rose NR, King HW, Blackledge NP, Fursova NA, Ember KJ, Fischer R, Kessler BM, Klose RJ (2016). RYBP stimulates PRC1 to shape chromatin-based communication between Polycomb repressive complexes. Elife.

[ref-36] Salgado E, Bian X, Feng A, Shim H, Liang Z (2018). HDAC9 overexpression confers invasive and angiogenic potential to triple negative breast cancer cells *via* modulating microRNA-206. Biochemical and Biophysical Research Communications.

[ref-37] Sanz H, Aponte JJ, Harezlak J, Dong Y, Ayestaran A, Nhabomba A, Mpina M, Maurin OR, Díez-Padrisa N, Aguilar R, Moncunill G, Selidji Todagbe A, Daubenberger C, Dobaño C, Valim C (2017). drLumi: an open-source package to manage data, calibrate, and conduct quality control of multiplex bead-based immunoassays data analysis. PLOS ONE.

[ref-38] Saunders PTK, Horne AW (2021). Endometriosis: etiology, pathobiology, and therapeutic prospects. Cell.

[ref-39] Saunders PTK, Whitaker LHR, Horne AW (2024). Endometriosis: improvements and challenges in diagnosis and symptom management. Cell Reports Medicine.

[ref-40] Sawa C, Yoshikawa T, Matsuda-Suzuki F, Deléhouzée S, Goto M, Watanabe H, Sawada J, Kataoka K, Handa H (2002). YEAF1/RYBP and YAF-2 are functionally distinct members of a cofactor family for the YY1 and E4TF1/hGABP transcription factors. Journal of Biological Chemistry.

[ref-41] Ściezyńska A, Komorowski M, Soszyńska M, Soszyńska M, Malejczyk J (2019). NK cells as potential targets for immunotherapy in endometriosis. Journal of Clinical Medicine.

[ref-42] Shannon P, Markiel A, Ozier O, Baliga NS, Wang JT, Ramage D, Amin N, Schwikowski B, Ideker T (2003). Cytoscape: a software environment for integrated models of biomolecular interaction networks. Genome Research.

[ref-43] Shin S, Chung YJ, Moon SW, Choi EJ, Kim MR, Chung YJ, Lee SH (2023). Single-cell profiling identifies distinct hormonal, immunologic, and inflammatory signatures of endometriosis-constituting cells. Journal of Pathology.

[ref-44] Takeuchi M, Matsuzaki K, Harada M (2024). Endometriosis, a common but enigmatic disease with many faces: current concept of pathophysiology, and diagnostic strategy. Japanese Journal of Radiology.

[ref-45] Taylor HS, Kotlyar AM, Flores VA (2021). Endometriosis is a chronic systemic disease: clinical challenges and novel innovations. Lancet.

[ref-46] Throwba HPK, Unnikrishnan L, Pangath M, Vasudevan K, Jayaraman S, Li M, Iyaswamy A, Palaniyandi K, Gnanasampanthapandian D (2022). The epigenetic correlation among ovarian cancer, endometriosis and PCOS: a review. Critical Reviews in Oncology/Hematology.

[ref-47] Viré EA, Mead S (2023). Gene expression and epigenetic markers of prion diseases. Cell and Tissue Research.

[ref-48] Wang T, Ji M, Sun J (2024). Identification and validation of an endoplasmic-reticulum-stress-related gene signature as an effective diagnostic marker of endometriosis. PeerJ.

[ref-49] Wang X, Zhang Z, Zeng W, Zhong Y, Xie D, Zhu W, Chen F, Du J, Zhang T (2022). Establishment of DNA methylation profile associated with TCM syndrome in endometriosis. Evidence-Based Complementary and Alternative Medicine.

[ref-50] Wu T, Hu E, Xu S, Chen M, Guo P, Dai Z, Feng T, Zhou L, Tang W, Zhan L, Fu X, Liu S, Bo X, Yu G (2021). clusterProfiler 4.0: a universal enrichment tool for interpreting omics data. Innovation.

[ref-51] Xu L, Wang J, Liu B, Fu J, Zhao Y, Yu S, Shen L, Yan X, Su J (2022). HDAC9 contributes to serous ovarian cancer progression through regulating epithelial-mesenchymal transition. Biomedicines.

[ref-52] Xu J, Yang T, Wu F, Chen T, Wang A, Hou S (2023). A nomogram for predicting prognosis of patients with cervical cerclage. Heliyon.

[ref-53] Yan Y, Jin X, Sun H, Pang S, Kong X, Bu J, Xu S (2021). MiR-139-5p targetedly regulates YAF2 and mediates the AKT/P38 MAPK signaling pathway to alleviate the metastasis of non-small cell lung cancer cells and their resistance against cisplatin. Cancer Management and Research.

[ref-54] Yang L, Pan X, Zhang Y, Zhao D, Wang L, Yuan G, Zhou C, Li T, Li W (2022). Bioinformatics analysis to screen for genes related to myocardial infarction. Frontiers in Genetics.

[ref-55] Yang S, Wang H, Li D, Li M (2024). An estrogen-NK cells regulatory axis in endometriosis, related infertility, and miscarriage. International Journal of Molecular Sciences.

[ref-56] Yoldemir T (2023). Evaluation and management of endometriosis. Climacteric.

[ref-57] Young VJ, Brown JK, Saunders PT, Duncan WC, Horne AW (2014). The peritoneum is both a source and target of TGF-β in women with endometriosis. PLOS ONE.

[ref-58] Yu X, Liu Y, Yin L, Peng Y, Peng Y, Gao Y, Yuan B, Zhu Q, Cao T, Xie B, Sun L, Chen Y, Gong Z, Qiu Y, Fan X, Li X (2019). Radiation-promoted CDC6 protein stability contributes to radioresistance by regulating senescence and epithelial to mesenchymal transition. Oncogene.

[ref-59] Zamurovic M, Tomic A, Djordjevic K, Simanic S, Sopta J, Rasulic L, Simic L, Jevtic J, Nedeljkovic-Arsenovic O, Rovcanin M (2023). Isolated deep infiltrating endometriosis of the sciatic nerve: a case report and overview of the literature. Medicina.

[ref-60] Zhang S, Zhang X, Guan X, Ma X, Chen H, Huang B, Chen D (2021). YAF2 exerts anti-apoptotic effect in human tumor cells in a FANK1- and phosphorylation-dependent manner. Biochemical and Biophysical Research Communications.

[ref-61] Zheng H, Liu X, Guo SW (2023). Corroborating evidence for aberrant expression of histone deacetylase 8 in endometriosis. Reproductive Medicine and Biology.

[ref-62] Zhou H, Xin Y, Li S (2023). A diabetes prediction model based on Boruta feature selection and ensemble learning. BMC Bioinformatics.

