# Supplementary figures and images for: Study on biomarkers associated with epigenetic factors in endometriosis combining transcriptome with experimental validation
Source: PeerJ. 2026 Feb 3;14:e20703. doi: 10.7717/peerj.20703 (PMC12880091; doi:10.7717/peerj.20703)

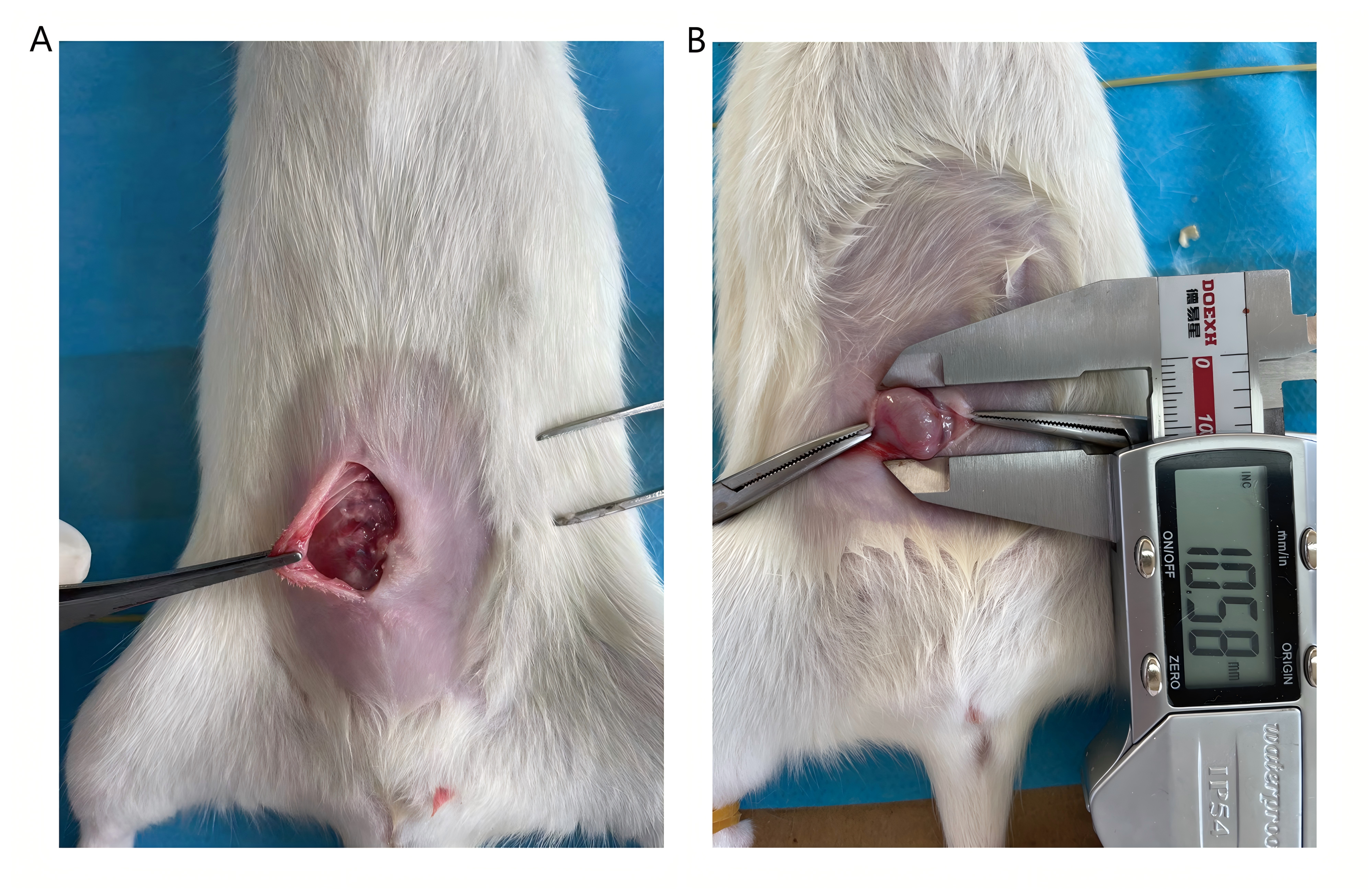

Supplement: Supplemental Information 1 — (A) Abdominal wall endometriotic lesions of model group rats. (B) Abdominal wall incisions without ectopic lesion of control group rats. [file peerj-14-20703-s001.jpg]

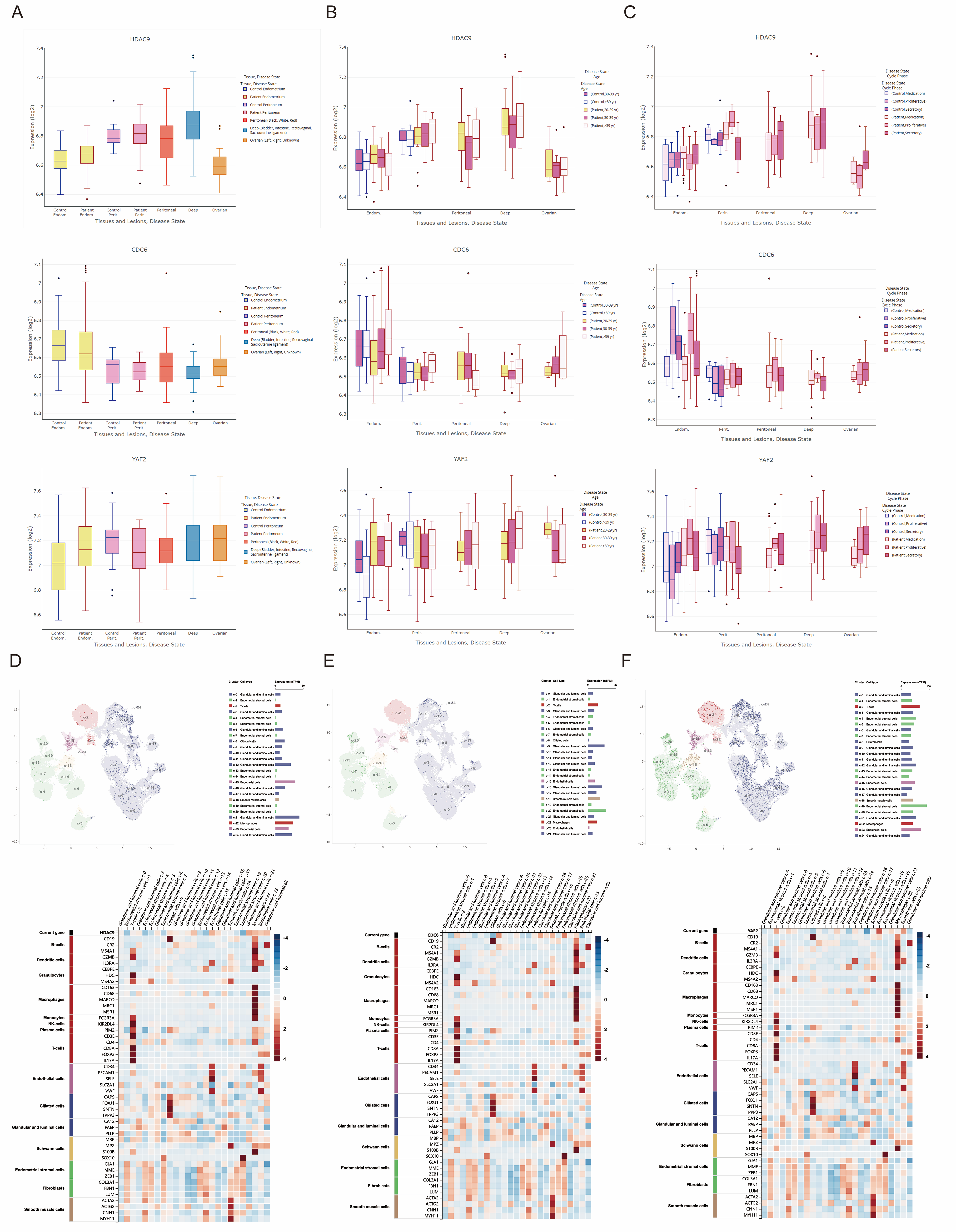

Supplement: Supplemental Information 2 — (A) Expression profiles of biomarkers across endometrial (disease/control), peritoneal (disease/control), and ovarian tissues: tissue status (x-axis) versus expression levels (y-axis, top to bottom: HDAC9, CDC6, YAF2). (B) Expression patterns of key genes across age-stratified tissue groups (x-axis: five tissue types) versus expression levels (y-axis, top to bottom: HDAC9, CDC6, YAF2). (C) Expression of biomarkers (top to bottom: HDAC9, CDC6, YAF2) across cell types, with darker shades indicating higher expression levels. (D–F) Cellular expression profiles of biomarkers HDAC9, CDC6, and YAF2. [file peerj-14-20703-s002.png]

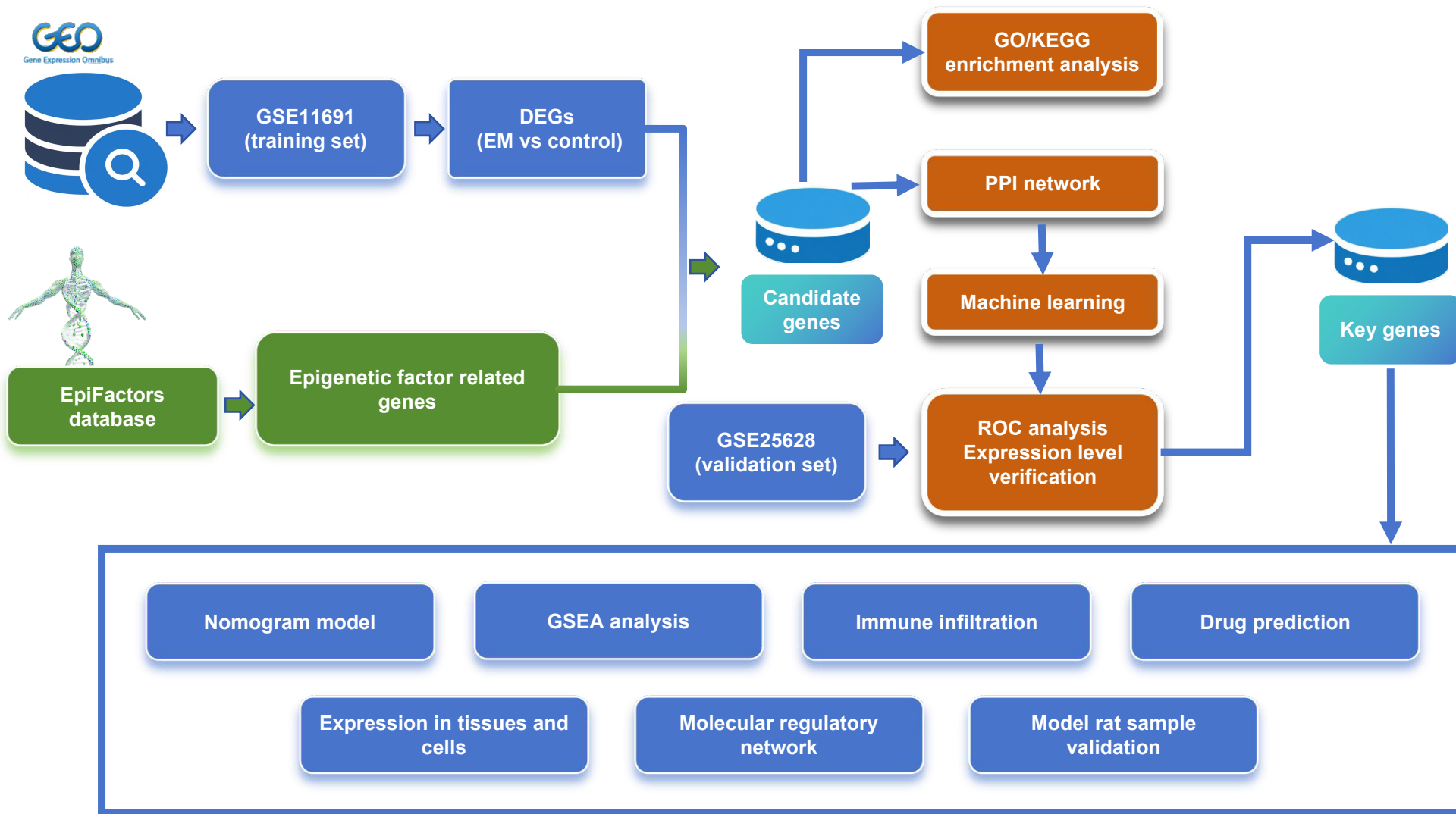

Supplement: Supplemental Information 12 — Visually illustrates the technical workflow of the present study. Candidate genes were screened by integrating the analysis of GEO dataset (GSE11691) and mining of the EpiFactors database; key genes were then identified following validation via machine learning and the GSE25628 dataset. Subsequent analyses (including nomogram modeling, immune infiltration, and drug prediction) were conducted, with final verification completed using a rat model. [file peerj-14-20703-s012.pdf]

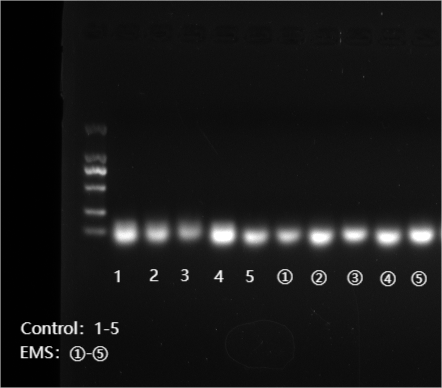

Supplement: Supplemental Information 15 [file peerj-14-20703-s015.zip › peerj-120698-Raw_Data_of_PCR/Raw Data of PCR/PCR/Raw PCR Gel Image/FigS3_Annotated_PCR_2024-11-20.png]

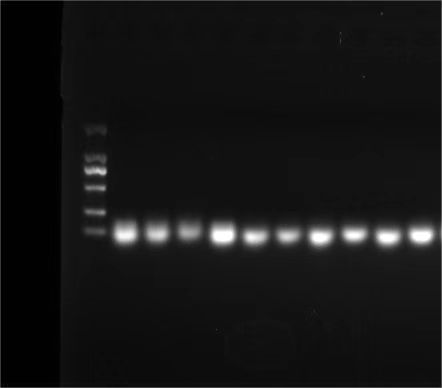

Supplement: Supplemental Information 15 [file peerj-14-20703-s015.zip › peerj-120698-Raw_Data_of_PCR/Raw Data of PCR/PCR/Raw PCR Gel Image/FigS3_Raw_PCR_2024-11-20.jpg]
